# Author Correction: High-fidelity parametric beamsplitting with a parity-protected converter

**DOI:** 10.1038/s41467-023-41822-5

**Published:** 2023-09-28

**Authors:** Yao Lu, Aniket Maiti, John W. O. Garmon, Suhas Ganjam, Yaxing Zhang, Jahan Claes, Luigi Frunzio, Steven M. Girvin, Robert J. Schoelkopf

**Affiliations:** 1 Departments of Applied Physics and Physics, Yale University, New Haven, 06511 CT USA; 2 Yale Quantum Institute, Yale University, New Haven, 06511 CT USA

**Keywords:** Quantum information, Qubits, Single photons and quantum effects, Quantum mechanics

Correction to: *Nature Communications* 10.1038/s41467-023-41104-0, published online 18 September 2023

In the “Acknowledgements” section of this article the grant number relating to Army Research Office (ARO) was incorrectly given as W911NF-22-1-0053 and should have been W911NF-23-1-0051. The original article has been corrected.

