# Supplementary material for: High-fidelity parametric beamsplitting with a parity-protected converter
Source: Nat Commun. 2023 Sep 18;14:5767. doi: 10.1038/s41467-023-41104-0 (PMC10507116; doi:10.1038/s41467-023-41104-0)
Supplement: Supplementary file 1 — Supplementary Information [file 41467_2023_41104_MOESM1_ESM.pdf]

Supplementary Information: High-fidelity parametric beamsplitting with a parity-protected converter

Yao Lu[1,2*†], Aniket Maiti[1,2*†], John W. O. Garmon[1,2], Suhas Ganjam[1,2], Yaxing Zhang[1,2], Jahan Claes[1,2], Luigi Frunzio[1,2], Steven M. Girvin[1,2] and Robert J. Schoelkopf[1,2*]

[1]Departments of Applied Physics and Physics, Yale University, New Haven, 06511, CT, USA.
[2]Yale Quantum Institute, Yale University, New Haven, 06511, CT, USA.
[1]Departments of Applied Physics and Physics, Yale University, New Haven, 06511, CT, USA.
[2]Yale Quantum Institute, Yale University, New Haven, 06511, CT, USA.

*Corresponding author(s). E-mail(s): physics.lu@yale.edu; aniket.maiti@yale.edu; robert.schoelkopf@yale.edu;
†These authors contributed equally to this work.

Supplementary Note 1 — Hamiltonian of the driven DC SQUID

The two-mode nature of a DC-SQUID has been well-studied in literature [1, 2]. In this section, we derive the joint Hamiltonian for these two modes in the presence of an AC-flux modulation. The SQUID circuit is shown in Fig. S1, where we include a small but finite linear inductance L_3 and capacitance C_3 that connects the two junctions. The flux variables across the junctions and the linear inductance are labeled as $\Phi_{1,2,3}$, where the first two directly correspond to the Josephson phases $\hat{\theta}_{1,2}$ in the main text. From this general formalism, we will derive the parity-protected Hamiltonian as a special case, with $\hat{\theta}_c$ and ϕ_d representing the coupler and actuator respectively. We will also derive the other limit of the common-driven SQUID and explicitly establish the Hamiltonian under a continuous spectrum of drive asymmetry.

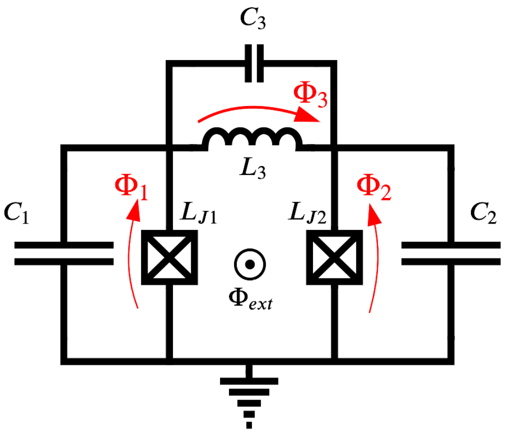

Fig. S1 The circuit diagram of a flux-driven SQUID.

We start with the general circuit Lagrangian for the driven SQUID in Fig. S1

$$\mathscr{L} = \frac{1}{2}\sum_{i=1}^{3} C_i \dot{\Phi}_i^2 + \sum_{i=1}^{2} E_{Ji}\cos\frac{\Phi_i}{\phi_0} - \frac{\Phi_3^2}{2L_3}, \quad (1)$$

with a constraint from the flux quantization relationship,

$$\Phi_1 - \Phi_2 + \Phi_3 = \Phi_{\text{ext}} + n\Phi_0, \tag{2}$$

where Φ_0 (ϕ_0) is the (reduced) flux quantum. Therefore, we may rewrite Eq. 1 as

$$\begin{aligned}\mathscr{L} &= \frac{1}{2}C_1\left(\dot{\Phi}_c + \dot{\Phi}_d\right)^2 + \frac{1}{2}C_2\left(\dot{\Phi}_c - \dot{\Phi}_d\right)^2 \\ &+ E_{J_1}\cos\frac{\Phi_c + \Phi_d}{\phi_0} + E_{J_2}\cos\frac{\Phi_c - \Phi_d}{\phi_0} \\ &+ \frac{1}{2}C_3\left(2\dot{\Phi}_d - \dot{\Phi}_{\text{ext}}\right)^2 - \frac{(2\Phi_d - \Phi_{\text{ext}})^2}{2L_3}.\end{aligned} \tag{3}$$

Here, we have defined the "differential mode" Φ_d, and the "common mode" Φ_c, as

$$\Phi_{c,d} = \frac{1}{2}\left(\Phi_1 \pm \Phi_2\right). \tag{4}$$

Under the assumption that the linear inductance L_{J3} and the capacitance C_{J3} are small, the differential mode frequency is much higher than that of the common mode. This means that, even under circuit asymmetry such that $C_1 \neq C_2$ and $E_{J_1} \neq E_{J_2}$, Φ_c and Φ_d remain good approximations for the normal-mode flux variables of the circuit. From this, the equation of motion for Φ_d can be approximated as

$$\begin{aligned}&\frac{d}{dt}\frac{\partial\mathscr{L}}{\partial\dot{\Phi}_d} - \frac{\partial\mathscr{L}}{\partial\Phi_d} \approx \\ &C_{d\Sigma}\ddot{\Phi}_d + L_{d\Sigma}^{-1}\Phi_d - 2C_3\ddot{\Phi}_{ext} - 2\frac{\Phi_{\text{ext}}}{L_3} = 0,\end{aligned} \tag{5}$$

where $C_{d\Sigma} = C_1 + C_2 + 4C_3$ and $L_{d\Sigma} = \left(L_{J1}^{-1} + L_{J2}^{-1} + 4L_3^{-1}\right)^{-1}$ are the total capacitance and inductance of the differential mode. When the drive frequency is much lower than the differential mode frequency, the steady state solution of Eq. 5 is given by

$$\langle\Phi_d\rangle \approx \Phi_{\text{ext}}/2. \tag{6}$$

This can also be seen from the fact that in Eq. 3, the last term is a high energy term that requires its numerator to be a "frozen" degree of freedom, $\left\langle 2\dot{\Phi}_d - \dot{\Phi}_{ext}\right\rangle$=0. Plugging this back in to Eq. 3 yields

$$\begin{aligned}\mathscr{L} &= \frac{1}{2}C_1\left(\dot{\Phi}_c + \frac{\dot{\Phi}_{ext}}{2}\right)^2 + \frac{1}{2}C_2\left(\dot{\Phi}_c - \frac{\dot{\Phi}_{ext}}{2}\right)^2 \\ &+ E_{J_1}\cos\frac{\Phi_c + \frac{\Phi_{\text{ext}}}{2}}{\phi_0} + E_{J_2}\cos\frac{\Phi_c - \frac{\Phi_{\text{ext}}}{2}}{\phi_0}.\end{aligned} \tag{7}$$

In reality, the differential flux modulation may also be contaminated by stray voltage coupling of the common mode to the flux line, as well as by the non-uniformity in the spatial distribution of the time-dependent flux that creates uneven voltage drops (electromotive forces) across the shunting capacitors (see Methods "Fine-tuning a differential drive" for a detailed analysis). Without loss of generality, the Hamiltonian can be written as

$$\begin{aligned}\hat{\mathcal{H}} &= 4E_C\hat{n}_c^2 + \epsilon(t)\,\hat{n}_c \\ &-E_{J_1}\cos\left(\hat{\theta}_c + \frac{\Phi_{\text{ext}}}{2\phi_0}\right) - E_{J_2}\cos\left(\hat{\theta}_c - \frac{\Phi_{\text{ext}}}{2\phi_0}\right),\end{aligned} \tag{8}$$

where $\epsilon(t)$ represents the effective common mode drive strength, and $\hat{n}_c$, $\hat{\theta}_c$ are the Cooper-pair number operator and the superconducting phase operator of the common mode, respectively. In the displaced frame, this Hamiltonian becomes (for symmetric SQUID of $E_{J_1} = E_{J_2} = E_J/2$)

$$\begin{aligned}\hat{\mathcal{H}}_{\text{disp}} &= 4E_C\hat{n}_c^2 + E_J\frac{\hat{\theta}_c^2}{2} \\ &- E_J\cos_{\text{NL}}\left(\hat{\theta}_c + \phi_1\right) - E_J\cos_{\text{NL}}\left(\hat{\theta}_c + \phi_2\right) \\ &= 4E_C\hat{n}_c^2 - E_J\cos\phi_c\cos\phi_d\cos\hat{\theta}_c \\ &+ E_J\sin\phi_c\cos\phi_d\sin\hat{\theta}_c - E_J\phi_c\hat{\theta}_c,\end{aligned} \tag{9}$$

where $\cos_{\text{NL}} x = \cos x + \frac{x^2}{2}$, and $\phi_{c,d} = \frac{1}{2}(\phi_1 \pm \phi_2)$ are the displacements in the phases of the common and the differential mode. From Eq. 8 and Eq. 9, we obtain these displacements as $\phi_d = \Phi_{\text{ext}}/2\phi_0$, and $\phi_c = \epsilon(t)\omega_d\hbar^{-1}/\left(\omega_d^2 - \omega_c^2\right)$, where ω_c and ω_d are the frequencies of the coupler and the drive. A pure differential drive corresponds to $\phi_c = 0$, where Eq. 9 reduces to

$$\hat{\mathcal{H}}_{DDS} = 4E_C\hat{n}_c^2 - E_J\cos\phi_d\cos\hat{\theta}_c, \tag{10}$$

which is the parity-protected Hamiltonian (Eq. 1) in the main text. In contrast, $\phi_d = 0$ happens for

a purely charge-driven transmon,

$$\hat{\mathcal{H}}_{trans} = 4E_C\hat{n}_c^2 - E_J\cos\phi_c\cos\hat{\theta}_c + E_J\sin\phi_c\sin\hat{\theta}_c - E_J\phi_c\hat{\theta}_c. \quad (11)$$

With Eq. 9, we can express the strengths of some of the parametric processes in terms of the displacement amplitudes, ϕ_c and ϕ_d. For example, the beamsplitter interaction arises from the $\cos\hat{\theta}_c$ modulation in the third line of Eq. 9. For purely RF drives where ϕ_d contains no DC component, both the beamsplitter rate and the Zeeman shift are proportional to $\phi_c^2+\phi_d^2$ under weak drive strengths. On the other hand, the last line of Eq. 9 gives rise to the odd parity interactions, with the leading order term being the '$ge/3$' interaction,

$$\hat{\mathcal{H}}_{ge/3} \approx E_J\left(\phi_c - \frac{\phi_c^3}{6}\right)\left(1-\frac{\phi_d^2}{2}\right)\hat{\theta}_c - E_J\phi_c\hat{\theta}_c \approx -\frac{E_J}{2}\phi_c\left(\phi_d^2+\frac{\phi_c^2}{3}\right)\theta_{c,\mathrm{zpf}}\left(\hat{c}^\dagger+\hat{c}\right), \quad (12)$$

which we used to experimentally characterize the residual drive asymmetry (Methods "Experimentally characterizing residual drive asymmetry").

Supplementary Note 2 — Parity protection and drive-frequency engineering

In this section, we discuss the importance of parity protection and drive-frequency engineering in the differentially-driven-SQUID (DDS), by contrasting it to a charge-driven transmon. In the latter's case (Eq. 11), only the 'even parity' part of the Hamiltonian ($\hat{\theta}_c^{2n}$, coming from the second term) that allows even numbers of mode quanta to interact, is useful for the bilinear beamsplitting process ($\propto \hat{\theta}_c^2$), The odd-parity terms ($\hat{\theta}_c^{2n+1}$, from the second line) introduce a set of nonlinear processes that are not only unhelpful, but can be actively harmful, leading to an incoherent excitation of the coupler that dephases the beamsplitter process, or an unintended exchange of sensitive quantum information with other modes in the system or the environment.

We are able to address both of these issues by utilizing the orthogonality of the coupler and actuator in the differentially-driven SQUID (DDS, Eq. 10), which offers a two-fold advantage through its parity protection and simpler drive engineering.

First, the forbidden linear coupling between the drive and the coupler avoids its displacement that converts dephasing into heating. This can be intuitively understood as high-frequency coupler dephasing combining with the drive to provide excitation at the coupler frequency. This process should be sensitive only to the spectrum of coupler frequency noise at the detuning between the drive and the coupler mode, and thus driving far-detuned from the coupler is also helpful.

Second, incoherent excitations may also result from driven resonant processes in combination with coupler decay. It is therefore imperative to avoid these resonances, the strongest of which is a squeezing of the coupler that is resonant near the coupler mode frequency (specifically, at half the g-f transition frequency). Intuitively, this can be thought of as a coherent excitation from the ground to the second excited state, combined with an incoherent decay back to the first excited state, thus leading to an over-all incoherent heating. This is a common issue in both the charge-driven transmon as well as the DDS, since squeezing and higher-order even-parity interactions are not forbidden by Eq. 10. However, in the DDS, the orthogonality between the actuator and the coupler greatly facilitates the engineering of the drives that are far-detuned from the coupler, suppressing both the squeezing and the dressed dephasing.

Finally, the selection rule enforced by the parity-protected Hamiltonian should forbid any odd-parity exchanges for modes participating in the couple, which is especially useful since strong drives can cause frequency shifts of the resonance condition. This includes resonances that could either cause undesired exchanges between the cavity ($\hat{a}$) and the coupler ($\hat{c}$), like $\phi_d\ \hat{a}\hat{c}^{\dagger 2}$+h.c. which was a limiting factor in [3], or resonances that cause direct excitations of the coupler through terms like $\phi_d^3\ \hat{c}^\dagger$ + h.c..

Which of these effects dominate in the experiment depends strongly on the frequencies of interest and the spectrum of environmental noise that couples to the system. Here, we take a broader approach by using numerical simulations to illustrate the clear difference between a common and a differential drive for a DC-SQUID, in the presence of one or both of the drive tones. We expect

the full dynamics in the presence of both cavities and a colored spectrum of environmental noise to be significantly more complicated.

Our simulations use well-established Floquet-Markov methods [4–6]. The Floquet basis provides an appropriate basis for analyzing the driven coupler, with the Floquet ground state smoothly mapping to the undriven ground state. Intrinsic coupler decay and dephasing can result in an effective excitation out of this ground state, represented by a 'hopping' rate to other Floquet states [6]. This can cause incoherent shifts in the beamsplitting strength and resonance condition, and thus dephase the beamsplitting process. Quantifying the coupler heating rate provides a simple way to gauge the unwanted effects of the drive, with the coupler excitation providing an estimate for the expected coupler-induced infidelity of the process.

We simulate the coupler in its undriven eigenbasis, with the first 50 eigenstates. We calculate the Floquet modes $|\alpha_n\rangle$, which for small amplitudes adiabatically map to the n-th undriven eigenstate (Fock state $|n\rangle$). We calculate the transition rates W_{mn} from $|\alpha_m\rangle$ to $|\alpha_n\rangle$ via

$$W_{mn} = \sum_{k=-\infty}^{+\infty} S_{FF}\left[\Delta_{nmk}\right] \times \left| \int_0^T \frac{e^{-ik\omega_d t}}{T} \langle \alpha_m\left(t\right) | \hat{O} | \alpha_n\left(t\right)\rangle dt \right|^2. \quad (13)$$

Here, ω_d is the greatest common divisor of the frequencies of the two drives, ω_{d1} and ω_{d2}, and $T = 2\pi/\omega_d$ is the least common multiplier of their periods. $\hat{O}$ (in the simulation taken to be the charge operator) is the system operator that is coupled to the bath, through $\hat{\mathcal{H}}_{int}/\hbar = \delta F\left(t\right)\hat{O}$, where the effect of bath is modelled by the noise parameter $\delta F\left(t\right)$. The fluctuations in this parameter are captured by $S_{FF}\left[\omega\right]$, the noise spectral density:

$$S_{FF}\left[\omega\right] = \int_{-\infty}^{+\infty} d\tau e^{i\omega\tau} \left\langle \delta F\left(\tau\right) \delta F\left(0\right)\right\rangle . \quad (14)$$

The argument of this spectral density in Eq. 13, $\Delta_{nmk} = \omega_m - \omega_n - k\omega_d$, represents all the possible frequencies at which the transition of interest is coupled to the bath.

We start with simulating the effects of dressed coupler dephasing, by looking at the lab-frame Hamiltonian of the coupler in the presence of both drive tones and incoherent coupler frequency fluctuations (Fig. S2a). We introduce a useful quantity, the drive asymmetry l,

$$l = \frac{|\phi_c|}{\sqrt{|\phi_c|^2 + |\phi_d|^2}}, \quad (15)$$

for which 0 corresponds to a fully differential drive, and 1 to a fully common mode drive. As a function of l, we calculate $W_{01} \times t_{\mathrm{SWAP}}$, which measures the coupler excitation after a single Alice-Bob swap operation. For a fair comparison, we also re-scale drive amplitudes to maintain a total beamsplitter rate of $g_{BS}/2\pi \approx$ 2MHz, corresponding to $t_{\mathrm{SWAP}} = 125$ns.

We find, as predicted by theory, that this effect is primarily sensitive to the noise spectral density of coupler frequency fluctuations ($S_{\omega_c\omega_c}[\Omega]$) at frequencies close to the detuning of the drive and the coupler mode ($\Omega \sim \Delta_{d_{1,2},c}$). In flux tunable devices, this noise spectrum is expected to decrease inversely with increasing frequency, suggesting that with a reasonable large drive detuning ($\sim$ 4.25 GHz for this experiment), the effect of flux-noise induced dressed dephasing should be negligible. We show here however, that that experimentally achievable drive symmetries suppress dressed dephasing by several orders of magnitude regardless of the dephasing rate (Fig. S2a).

Through a similar Floquet-Markov calculation with both tones, we also calculate an effective coupler heating rate arising from a dressing of intrinsic coupler decay (Fig. S2b), specifically for the differentially driven Hamiltonian. Here, we find that despite the parity protection, there exists a significant heating rate within $\sim$ 1 GHz of the resonant squeezing, which occurs near the coupler frequency. This heating can intuitively be understood as a combination of a $|g\rangle \rightarrow |f\rangle$ coherent transition due to squeezing, and a $|f\rangle \rightarrow |e\rangle$ incoherent transition due to coupler decay. Choosing a large drive-detuning strongly suppresses the squeezing strength and avoids this issue. We also find weaker resonances at frequencies that are at a half ($\phi_d^4 c^{\dagger 2}$) and a third ($\phi_d^6 c^{\dagger 2}$) of the resonant squeezing process, but we are able to utilize the freedom in choosing our drive frequencies to evade them.

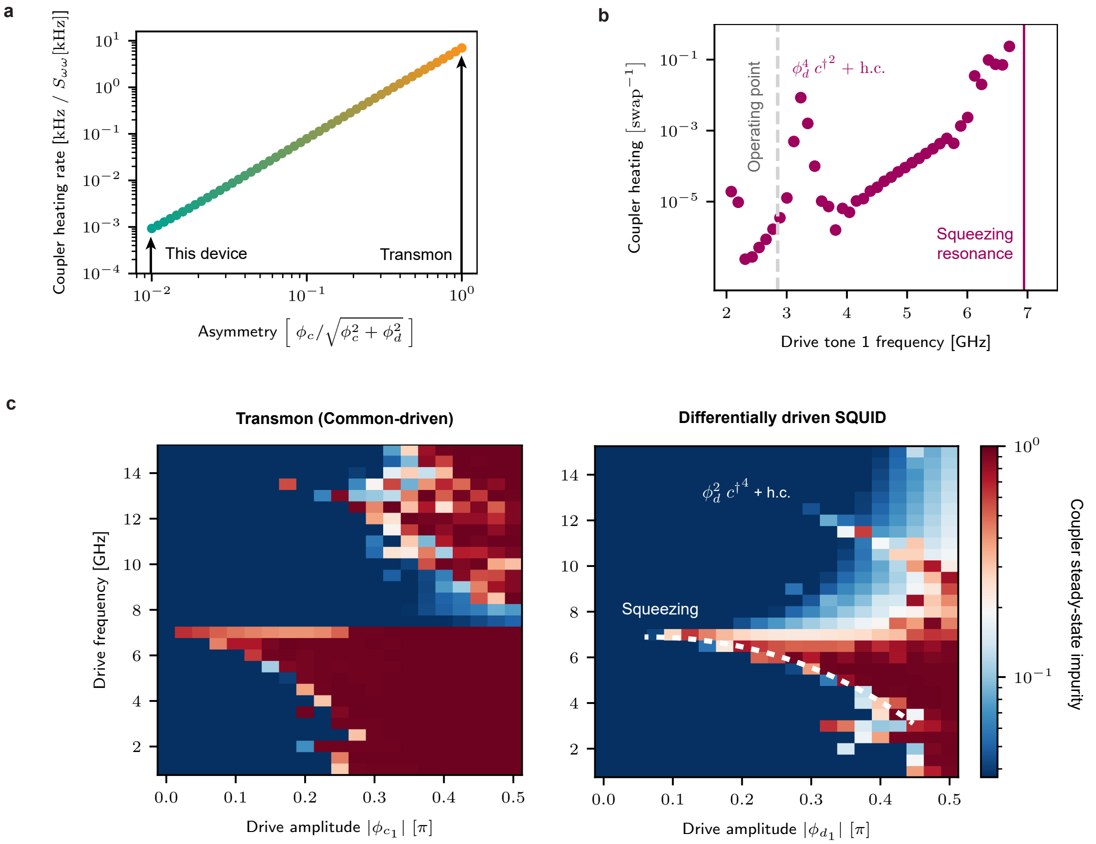

Fig. S2 Floquet simulations demonstrating advantages of the differentially-driven SQUID. a, The heating rate of the coupler from the Floquet ground state to the Floquet excited state, as a result of two drive tones ($\omega_{d_1} = 2\pi\times 2.772$GHz, $\omega_{d_2} = 2\pi \times 3.003$GHz) with various amount of drive asymmetry. Plotting the heating rate normalized by the spectral density of the dephasing $S_{\omega_c\omega_c}[\Delta_{d_2,c}]$ reveals the strong dependence of the coupler heating on the drive asymmetry, with a suppression by several orders of magnitude as we move from large asymmetries (orange) to small asymmetries (teal) that are practically achievable (the black arrows indicate the value of asymmetry in this experiment, and in the case of a single-junction transmon, respectively). **b**, A two-tone Floquet-Markov simulation illustrating the choice of drive frequencies for the differentially driven SQUID, at a fixed drive amplitude. The driven coupler heating rate is plotted (magenta) as a function of frequency of drive tone 1 (with the other drive frequency fixed to satisfy the beamsplitter resonance condition), in the presence of intrinsic coupler decay. We see that even for a purely differential drive, the amplitude-dependent squeezing (here resonant at $\omega_{gf/2} = 6.94$ GHz) can convert decay into heating (magenta). We place the drives far red-detuned from the coupler (grey dashed line), avoiding both the coupler squeezing and weaker nearby resonances, like the $\phi_d^4\,\hat{c}^{\dagger 2}$ + h.c. process. **c**, The impurity of the driven steady-state of the coupler, under a single drive tone, for the common and differentially driven cases. The blue and white regions imply a cold and hot coupler respectively, while the red regions are a result of chaotic behaviour or coupler ionization. The differentially-driven SQUID shows a significantly larger available drive space, where one can drive at higher amplitudes at almost every drive frequency without ionizing the coupler. Some residual limiting features in this plot are also clearly recognizable, like coupler squeezing (white dashed line is a guide to the eye), and the $\phi_d^2\hat{c}^{\dagger 4}$ + h.c. process, both of which follow a downward trend due to the Zeeman shift.

Finally, to provide an intuition for the combined effect of the odd-order processes forbidden by the parity protection, we show the marked difference in the allowed 'drive space' for the common and the differentially driven SQUID (Fig. S2c). We define the drive space to be the allowed frequencies and amplitudes of a single drive tone that can drive the converter while keeping the coupler in its driven ground state. Since a large portion of either plot shows chaotic behavior and coupler

ionization [7], we use the impurity of the steady-state density matrix as our performance metric, which circumvents the need for a precise mapping between the Floquet states and the undriven states. Since these are steady state calculations, the impurity of the coupler in regions where it is not ionized should not directly be interpreted as the heating rate for short time scales. We observe that the differential drive's allowed frequency and amplitude range is much larger than those allowed for the common drive, clearly demonstrating the former's advantage in this simplistic scenario. This simulation only contains a single drive tone and simulates just the coupler mode, but we expect this advantage in drive space to hold even in the presence of the coupled storage modes and multiple drives, since a large fraction of the additional parasitic processes will also be forbidden for the parity-protected Hamiltonian.

Supplementary Note 3 — Suppressing the back-action of the buffer cavity

While we have discussed the elimination of a linear coupling between the buffer mode and the coupler in our Methods, quantum fluctuations in the buffer mode might still dephase the coupler even in the presence of a purely non-linear coupling. This can be seen by writing the buffer-SQUID Hamiltonian in the dressed ladder-operator basis,

$$\begin{aligned}\hat{\mathcal{H}}/\hbar &= \omega_c\hat{c}^\dagger\hat{c} + \omega_B\hat{B}^\dagger\hat{B}\\ &- \frac{E_{J_1}}{\hbar}\cos_{\rm NL}\left(\hat{\theta}_c + k\hat{\theta}_B\right) - \frac{E_{J_2}}{\hbar}\cos_{\rm NL}\left(\hat{\theta}_c - k\hat{\theta}_B\right)\\ &\approx \omega_c\hat{c}^\dagger\hat{c} + \omega_B\hat{B}^\dagger\hat{B} - \chi_{Bc}\hat{B}^\dagger\hat{B}\hat{c}^\dagger\hat{c}. \end{aligned} \tag{16}$$

Here, ω_B, $\hat{B}$ and $\hat{\theta}_B$ are the frequency, the ladder operator, and the phase operator of the buffer mode. $k = \frac{1}{2}M_{Bc}L_B^{-1}$ measures the participation of the buffer-mode phase in the superconducting phases across the SQUID junctions, with L_B and M_{Bc} being the self-inductance of the buffer mode and the mutual inductance between the buffer mode and the SQUID loop, respectively. The cross-Kerr term emerges from the differential coupling with a strength of $\chi_{Bc} \approx E_J\theta^2_{c,\rm zpf}\theta^2_{B,\rm zpf}k^2$, and translates the photon-number fluctuations in the buffer mode to coupler dephasing noise. For thermal-photon distribution and coherent-photon distribution, the dephasing rates are given by [8]

$$\begin{aligned}\Gamma_\phi^{\rm th} &= \chi^2_{Bc}\frac{\overline{n}_{\rm th}}{\kappa_B},\\ \Gamma_\phi^{\rm coh} &= \chi^2_{Bc}\frac{\overline{n}_{\rm coh}\frac{\kappa_B}{2}}{(\omega_d-\omega_B)^2 + \left(\frac{\kappa_B}{2}\right)^2},\end{aligned} \tag{17}$$

where $\overline{n}_{\rm th}$ and $\overline{n}_{\rm coh}$ represent the mean thermal-photon number and coherent-photon number of the buffer mode, respectively. On the other hand, the beamsplitter rate is only linearly dependent on the cross-Kerr strength, $g_{BS} \propto \chi_{Bc}\overline{n}_{\rm coh}$. This allows us to suppress these dephasing rates while maintaining the beamsplitting rate, simply by reducing the cross-Kerr strength, and increasing the buffer drive strength (to increase $\overline{n}_{\rm coh}$). We calculated using a HFSS-EPR simulation [9] that our design has $\chi_{Bc} \approx 2\pi \times 0.1$kHz. which corresponds to negligible dephasing rates of $\Gamma_\phi^{\rm th} \ll 1$Hz, $\Gamma_\phi^{\rm coh} \ll 1$kHz. With a drive power of $P \approx -50$dBm at the buffer cavity drive port, we can realize a differential drive amplitude of $\phi_d \approx 0.2\Phi_0$, that drives a sufficiently fast beamsplitter for this experiment.

Supplementary Note 4 — Decoherence in the dual-rail subspace

In this section, we derive the evolution of the dual-rail density matrix (Methods Eq. 24), and discuss how the dual-rail decay and dephasing rates (κ_1 and κ_φ) are related to fluctuations in the undriven system. Without loss of generality, the rotating frame Hamiltonian of the driven dual-rail qubit is given by

$$\begin{aligned}\hat{\mathcal{H}}(t)/\hbar &= \left(g_{\rm BS} + \delta g(t)\right)\left(\hat{a}^\dagger\hat{b} + \hat{a}\hat{b}^\dagger\right)\\ &+ \delta\omega_a(t)\hat{a}^\dagger\hat{a} + \delta\omega_b(t)\hat{b}^\dagger\hat{b}\\ &+ \delta f_a(t)\hat{a}e^{-i\omega_a t} + \delta f_b(t)\hat{b}e^{-i\omega_b t} + h.c.\end{aligned} \tag{18}$$

Here, δg represents the fluctuation of the beamsplitter rate, which could arise from a dispersion of beamsplitting strength with respect to beamsplitting strength. $\delta\omega_{a,b}$ are the frequency fluctuations of the Alice and Bob cavities, which could arise from their cross-Kerr to the coupler mode, or from sources like a hot ancilla or other intrinsic dephasing. Imperfections in control electronics,

including slower drifts over time, could also contribute to these amplitude or frequency fluctuations. Finally, $\delta f_{a,b}$ are the cavities' couplings to environmental decay channels. These noises ($\delta x = \{\delta g, \delta\omega, \delta f\}$) are characterized by their spectral density (Eq. 14), which are related to the decay and dephasing rates of the individual cavities,

$$\kappa_{\downarrow}^{a,b} = S_{f_{a,b}f_{a,b}}[\omega_{a,b}],\ \kappa_{\phi}^{a,b} = S_{\omega_{a,b}\omega_{a,b}}[0]/2. \quad (19)$$

To analyze how Eq. 18 gives rise to decoherence in the dual-rail subspace, we work in the eigenbasis of the beamsplitter interaction, $|\text{Alice}, \text{Bob}\rangle = |00\rangle$, $|\psi^{\pm}\rangle = \left(|01\rangle \pm |10\rangle\right)/\sqrt{2}$, and $|11\rangle$, and rewrite this Hamiltonian in the matrix form,

$$\hat{\mathcal{H}}(t)/\hbar = \begin{bmatrix} 0 & \delta f_-(t) & \delta f_+(t) & 0 \\ \delta f_-(t)^* & \delta\Omega_-(t) & \frac{\delta\omega_-(t)}{2}e^{-2ig_{\text{BS}}t} & -\delta f_-(t) \\ \delta f_+(t)^* & \frac{\delta\omega_-(t)^*}{2}e^{2ig_{\text{BS}}t} & \delta\Omega_+(t) & \delta f_+(t) \\ 0 & -\delta f_-(t)^* & \delta f_+(t)^* & \delta\omega_+(t) \end{bmatrix}, \quad (20)$$

where

$$\delta\Omega_{\pm} = \delta\omega_a(t)/2 + \delta\omega_b(t)/2 \pm \delta g(t),$$
$$\delta f_{\pm} = \left(\delta f_a(t)e^{-i\omega_a t} \pm \delta f_b(t)e^{-i\omega_b t}\right)\frac{e^{\mp ig_{\text{BS}}t}}{\sqrt{2}},$$
$$\delta\omega_{\pm} = \delta\omega_a(t) \pm \delta\omega_b(t).$$

This allows the calculation of the transition rates among the four eigenstates using time-dependent perturbation theory [10], from which we obtain the decay rates from $|\psi^{\pm}\rangle$ to $|00\rangle$ as $\kappa_{\psi^{\pm}} = \left\{S_{f_af_a}[\omega_a \pm g_{\text{BS}}] + S_{f_bf_b}[\omega_b \pm g_{\text{BS}}]\right\}/2$. The relaxation between $|\psi_{\pm}\rangle$ due to the cavity frequency fluctuation $\delta\omega$, as well as the beamsplitter rate fluctuation δg, lead to dephasing in the dual-rail subspace at a rate $\kappa_{\varphi} = \left\{\bar{S}_{\omega_a\omega_a}[2g_{\text{BS}}] + \bar{S}_{\omega_b\omega_b}[2g_{\text{BS}}]\right\}/4 + S_{gg}[0]$, with $\bar{S}[\omega] = \left\{S[\omega] + S[-\omega]\right\}/2$ being the symmetrized spectral density. At small g_{BS} and weak dissipation rates, and assuming the cavity frequency fluctuations are uncorrelated, one can see how these rates are related back to cavity decay and dephasing rates, $\kappa_{\psi^{\pm}} = \kappa_1 = (\kappa_{\downarrow}^a + \kappa_{\downarrow}^b)/2$, $\kappa_{\varphi} = (\kappa_{\phi}^a + \kappa_{\phi}^b)/2 + S_{gg}[0]$.

From here, it is straightforward to write down the evolution of the dual-rail qubit's density matrix, in the basis of $|\psi^{\pm}\rangle$,

$$\rho_{|\psi^{\pm}\rangle}(t) = \frac{1}{2}e^{-\kappa_1 t}\begin{bmatrix} 1 & e^{i\Omega t - \kappa_{\varphi}t} \\ e^{-i\Omega t - \kappa_{\varphi}t} & 1 \end{bmatrix}, \quad (21)$$

where $\Omega = 2g_{\text{BS}}$. This can be easily transformed to $|10\rangle$ and $|01\rangle$ basis (denoted as ρ_{DR}),

$$\rho_{\text{DR}}(t) = \frac{1}{2}e^{-\kappa_1 t}\times \begin{bmatrix} 1 + e^{-\kappa_{\varphi}t}\cos(\Omega t) & i\sin(\Omega t)\,e^{-\kappa_{\varphi}t} \\ -i\sin(\Omega t)\,e^{-\kappa_{\varphi}t} & 1 - e^{-\kappa_{\varphi}t}\cos(\Omega t) \end{bmatrix}, \quad (22)$$

which becomes Eq. 24 in Methods.

Supplementary Note 5 — Limits on performance from coupler-storage sideband collision

We now provide a hypothesis for the limitations on fidelity and achievable beamsplitting strength that we find in our device. We see in experiment (Methods Fig. 7) that the effective beamsplitter decoherence rate remains stable as a function of drive amplitude, until it suddenly rises at $|\phi_d| \sim 0.13\pi$. This is accompanied by a super-quadratic dependence of g_{BS} on the drive amplitude. A hypothesis for this effect is an inflation of the interaction between the cavities and the coupler, mediated by the beamsplitter drives. This could arise from a drive-induced coupler-cavity sideband interaction, that is then magnified by the coupler's Zeeman shift in the regime of strong drives. Here, we investigate the strength of such a 'sideband collision', first focusing on Bob, which is closer in frequency to the coupler. By combining Eqs. 11,12, and 15 in the main text, one obtains a photon exchange interaction between the coupler and Bob in the rotating frame:

$$\frac{\hat{\mathcal{H}}_{bc}^{(\text{RWA})}}{\hbar} \approx \sum_{n=1}^{\infty}\omega_c J_n\left(|\phi_{d_1}|\right)J_n\left(|\phi_{d_2}|\right)\cos\left(n\Delta_d t\right) \times \beta_b\left(e^{i\Delta_{bc}t}\,\hat{b}^{\dagger}\hat{c} + \text{h.c.}\right). \quad (23)$$

Here, $\Delta_{bc} = \omega_b - \omega_c + \omega_{\text{Z},b} - \omega_{\text{Z},c}$ represents the detuning between Bob and coupler after accounting for Zeeman shifts. When the modulation frequency satisfies $\Delta_d = \Delta_{bc}/n$, a resonant 'iSWAP'

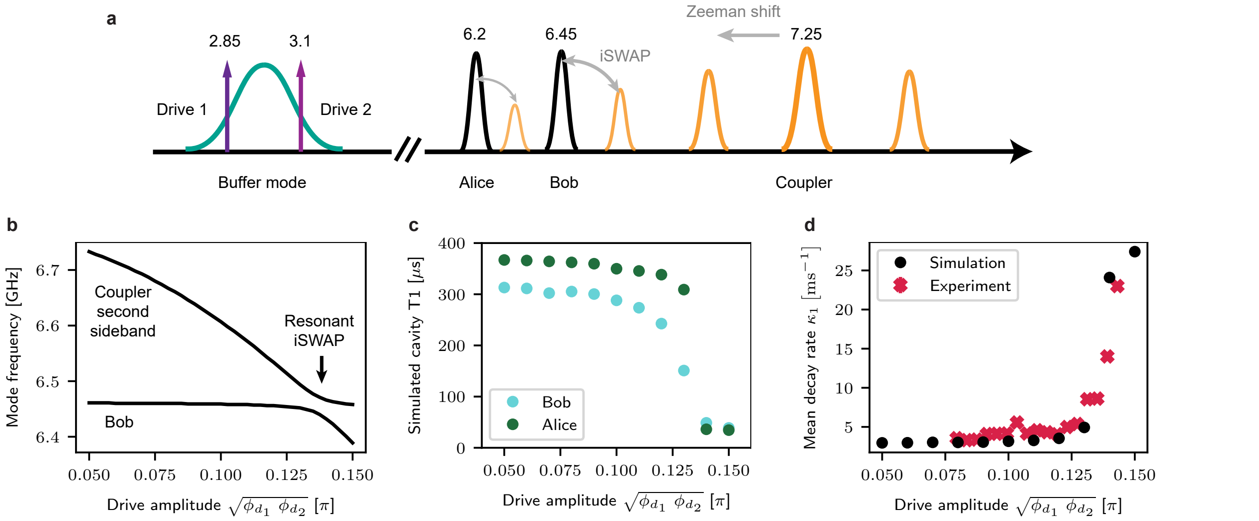

Fig. S3 Coupler-cavity sideband collision on increasing drive amplitude a, Frequency stack of the driven system. Due to the Zeeman shift, the coupler frequency is pushed towards the Alice and Bob cavities. The parametric modulation also creates sidebands of the coupler that are spaced by the two-tone detuning. These sidebands are coupled to the cavities through detuned iSWAP interactions, that get closer to resonance at stronger drive amplitudes. **b**, Simulation of the frequencies of the Bob cavity and the second (red-detuned) sideband of the coupler as a function of the drive amplitude. A clear avoided crossing is seen around drive amplitude of 0.14π when the coupler sideband collides into Bob, corresponding to a resonant iSWAP interaction. **c**, The Floquet-Markov simulations showing the reduction of the cavities' lifetimes with respect to the drive amplitude. Alice's and Bob's T_1s are obtained from two separate simulations, where only Alice or Bob mode is included along with the coupler, respectively. In both simulations, decay channels were set to produce similar undriven lifetimes of Alice, Bob and the coupler to the experimental values. **d**, A comparison of the mean of the decay rates (black circles) obtained from the simulation in **c** to the experimentally obtained decay rate of the dual-rail qubit (red crosses, same as main text Fig. 7). The curves show qualitative agreement, but the simulations only capture processes that involve the individual cavities and the coupler, and therefore a quantitative agreement is not expected.

interaction occurs between Bob and the coupler, which we interpret as the n-th sideband collision. This can be intuitively understood as a sideband interaction [11], as illustrated in Fig. S3a. The two-tone flux modulation creates coupler sidebands spaced by the tone detuning Δ_d which, when pushed down by the Zeeman shift, start interacting strongly with the storage cavities. We identify from Floquet simulations that around a drive amplitude of 0.14π, the second sideband of the coupler crosses the Bob mode (Fig. S3b), thereby limiting Bob's lifetime due to an inherited decay. We expect Alice to show similar behaviour, encountering the coupler's third sideband at a similar Zeeman shift, but we expect this process to be higher order and therefore weaker.

To study the implications of this increased hybridization, we perform Floquet-Markov simulations to find predicted T_1's for both cavities as a function of drive strength (Fig. S3c). These decay rates are extracted from two separate simulations, with each simulation including the coupler mode and only a single cavity mode, but should be sufficient to capture the iSWAP interactions. We therefore do not necessarily expect the simulations to show quantitative agreement with experiment, as the experiment could be limited by processes that simultaneously involve both cavities, or the coupler's readout mode. However, for a qualitative test of this hypothesis, we compare the predicted mean cavity decay rates κ_1 to those found in experiment (main text Fig. 7), and find a reasonable agreement (Fig. S3d). This issue of increased hybridization could potentially be avoided by redesigning the coupler driven frequency at the operating point, for example by placing it red-detuned to the cavities, such that the cavities are limited by a higher-order sideband.

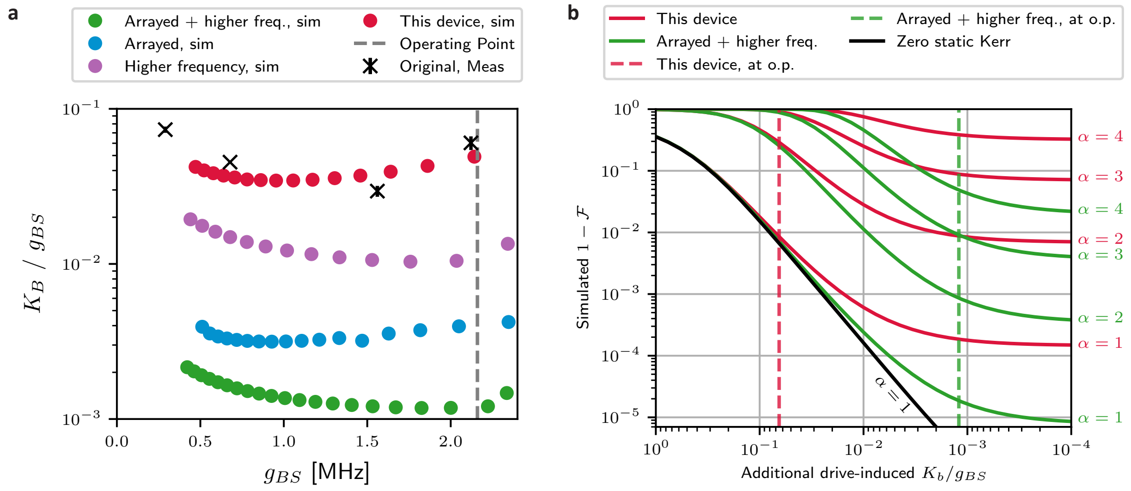

Fig. S4 Drive-induced cavity Kerr. a,The ratio between the beamsplitter rate, g_{BS}, and the self-Kerr of Bob, K_B, plotted against the beamsplitter rate. In the original circuit (red dots and blue crosses), this ratio is limited by the Zeeman shift as well as the sideband collision issue. By rearranging the frequency stack such that the coupler is further detuned from the cavities (f_c =7.75GHz, adjusting g_{ac} and g_{bc} accordingly to maintain the coupler-cavity hybridization), this ratio increases by almost a factor of 5 (orange dots), at the operating point where $g_{BS}/2\pi \approx$ 2MHz. Further optimization is possible by arraying the device into 3 SQUIDs (green dots), which roughly increases this ratio by a factor of $3^2 = 9$. **b**, Swap operation infidelity for various coherent states as a function of drive-induced Kerr, at fixed $g_{BS}/2\pi = 2.16$MHz. Static Kerr is set to be the same as the current device (red), and the arrayed device with 3 SQUID (blue). Cavity Kerr leads to infidelity through both an undesired photon-number-dependent phase accumulation as well as a shift in the beamsplitting resonance condition for each photon manifold. When $K_b/g_{BS} \sim 1$, this amounts to photon exchange only occurring in a single photon manifold. As K_b/g_{BS} is reduced the swap fidelity of coherent states reduces quadratically with this ratio (highlighted by the black line corresponding to zero static Kerr), until it saturates to an infidelity limit imposed by $K_b^{\text{static}}/g_{BS}$. The red and green dashed lines indicate the drive-induced K_b/g_{BS} of the current device, and the arrayed SQUID at higher frequency, respectively.

Supplementary Note 6 — Cavity nonlinearity induced by the driven SQUID

In addition to the increased cavity decay rates due to the sideband collision, large drive amplitudes on the SQUID coupler may also induce more nonlinearity to the cavities. We investigate the magnitude of this induced nonlinearity, it's effect on the beamsplitter, and strategies to mitigate the driven nonlinearity in this section.

A natural contribution to the static Kerr of the cavities comes from the SQUID coupler, which is in the transmon regime and has a self-Kerr of $K_c/2\pi \approx 125$MHz. As a result of the linear dispersive coupling, the storage cavities weakly hybridize with the coupler and inherit self-Kerrs, $K_{a(b)} \approx \left(\frac{g_{a(b)c}}{\Delta_{a(b)c}}\right)^4 K_c$. We directly measure this self-Kerr by first displacing the cavity to $|\alpha\rangle$, followed by a delay time of t that incurs a phase space rotation proportional to $\Delta + \alpha^2 \frac{K_b}{2}$, where Δ is the detuning between the cavity and drive frequencies. We then use a second displacement pulse with opposite phase that attempts to bring the cavity back to vacuum, with full revival determined by Δ, K_b and t. By performing a measurement conditioned on the cavity being in the vacuum state using the ancilla qubit and repeating this measurement as a function of α, we are able to quantify this revival and extract both the Kerr and detuning [12].

The cavity self-Kerrs are measured to be $K_a/2\pi \approx$ 5kHz and $K_b/2\pi \approx$ 15kHz when the coupler is undriven. When the coupler is driven, the Zeeman shift pushes the coupler frequency lower, bringing it closer to the cavities and therefore imposing a higher amount of nonlinearity to the cavities. This is accompanied by the sideband collision discussed in the previous section, which also causes a stronger hybridization between the coupler and the cavities. At the operating point where a beamsplitter rate of $\sim$ 2.2MHz is achieved, we measure the self-Kerr of Bob cavity to be around 128kHz. We expect the self-Kerr

of Alice to remain lower than Bob's, since the sideband it encounters is higher-order. While this aggravated nonlinearity is relatively harmless in the dual-rail subspace central to our investigation, it could introduce a coherent error in beamsplitter operations that involve large photon numbers, and optimizing the DDS' performance by reducing the drive-induced Kerr is an important future direction.

There exist multiple solutions that are fully compatible with our architecture, as demonstrated in Fig. S4. For instance, since the dominant source of cavity nonlinearity is from the Zeeman shift and the sideband collision, we could rearrange our mode frequencies such that the coupler is detuned further from the cavities. Thus, at the same drive amplitude (that produces the same g_{BS}), both the coupler and its sideband stay further away from the cavities, which is helpful for decreasing the Kerr/g_{BS} ratio. We could also redesign the coupler as an array of dc SQUIDs, which would suppress the coupler Kerr by a factor N^2, where N is the number of the SQUID loops. Finally, the cavity Kerrs can also be dynamically canceled through additional microwave drives on the coupler, as has been shown in [13], or corrected via additional unitaries like SNAP [12].

To place the quantity g_{BS}/K_b into context, and to get a sense of how significantly this nonlinearity affects higher-photon manifolds, we examine the limits on swap fidelity imposed by this coherent error. Using time-domain QuTiP [14] simulations, we first calibrate a swap pulse at our operating point of $g_{BS}/2\pi = 2.16$ MHz optimized for the single-photon manifold, as done in experiment. We then apply this pulse to a coherent state in the presence of both static and drive-induced Kerr (scaling all driven Kerrs proportionally), computing the final state fidelity $|\langle\psi_{ideal}|\psi_{final}\rangle|^2$ to the ideal swapped coherent state $|\psi_{ideal}\rangle$.

Fig. S4b shows this swap infidelity as a function of drive-induced K_b/g_{BS} for different coherent states and static Kerrs. When the drive-induced Kerr is on the order of the beamsplitting rate, the coherent state swap fidelity suffers drastically as the beamsplitter becomes increasingly off-resonant for all but the single-photon manifold. As the driven Kerr is reduced, the infidelity reduces quadratically for decreasing K_b/g_{BS} until eventually saturating to a background infidelity limit imposed by the static Kerr.

These simulations demonstrate that by implementing modifications compatible with our design, we can achieve a substantial improvement in performance. Arraying the coupler ($N = 3$) and shifting to a higher frequency would lead to a Kerr-limited swap fidelity of more than 99.9% for the $|\alpha = 2\rangle$ coherent state, and above 99% for $|\alpha = 3\rangle$. Note that this effect could be mitigated to some extent by calibrating the beamsplitter in photon manifold corresponding to the mean coherent state photon number. While applications with much more stringent linearity requirements exist [15, 16], significant progress towards realizing hardware-efficient error correction has been made with protocols using states with mean photon numbers as low as the states simulated here [17–19]. However for most applications to high fidelity control of logical qubits, including an additional unitary resolving this coherent error will be useful.

Supplementary Note 7 — DC flux calibration and junction E_J asymmetry

Operating at the DC flux sweet spot of the SQUID is not only desirable for combating flux noise, but is also critical to keeping our aforementioned parity-protection. To ensure that we operate at $\Phi_{\mathrm{DC}} = 0$, we have a coil built in to our package to apply a static magnetic field through the SQUID loop. This coil is made out of thin NbTi wire wrapped around a copper spool, similar to the magnet in many resonant JPA designs, and is placed sufficiently far from the converter and cavities, so as to not spoil any high-Q modes. The coil is connected to a DC power supply, where we can tune the source Voltage as desired.

The spectroscopy of the coupler readout serves as an initial estimate of the zero flux point, where we observe a periodic tuning of the coupler readout frequency as the DC source is swept, due to the repulsion of the coupler mode. (Fig. S5a).

To obtain a more precise and more direct measurement of the zero-flux point, we then perform coupler spectroscopy while sweeping the now-calibrated DC source (Fig. S5b). The coupler frequency is measured near zero flux as well as further on the slope. As the coupler frequency sweeps into resonance with Alice and Bob, the modes strongly hybridize, obscuring the coupler resonance. The measured coupler frequency is well

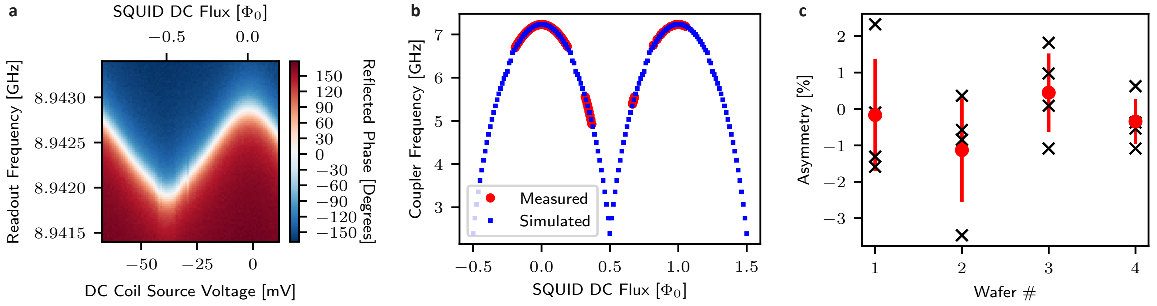

Fig. S5 DC flux and junction asymmetry. a, Spectroscopy of the coupler readout resonator while tuning source voltage of the DC flux coil. This curve provides an initial estimate of the zero flux point as well as the conversion between coil voltage and Flux Quantum. **b,** Coupler frequency vs. DC flux. The data is fit to a model that takes both linear inductance of the SQUID loop and hybridization between coupler, Alice, and Bob into account. **c,** Junction asymmetry as backed out from room temperature resistance measurements from SQUID devices on multiple wafers. These devices were fabricated using the same methods as (and contemporaneous with) the device demonstrated in the main text (Supplementary Note Supplementary Note 11).

fit to a model that takes both the linear inductance in the SQUID loop and the hybridization with Alice and Bob into account, yielding the simulated points in Fig. S5b.

Another requirement of the differentially-driven Hamiltonian (Eq. 1 in the main text) is that the Josephson energies of the two junctions in the SQUID are the same, which in the practical sense means the junctions should have identical size. While this is the case in design, imperfections in the fabrication processes, especially in the e-beam writing step, results in an inevitable deviation of the E_J of the two junctions. To quantify the typical value of the E_J asymmetry, we measure the room-temperature resistance of the Josephson junctions, which is related to their Josephson energies through the Ambegaokar-Baratoff relation [20]. Through this way, we measured the E_J asymmetry, which we define as $(E_{J_1} - E_{J_2})\,/\,(E_{J_1} + E_{J_2})$, for the SQUID devices on four different wafers that were fabricated around the same time (Fig. S5c). From these measurements, We observe a negligibly small E_J asymmetry of 0.3%±1.3%.

Supplementary Note 8 — Calibrating a beamsplitter pulse

In order to calibrate the beamsplitter that is used in the RB experiment, we must accurately tune up the pulses on the RF controls. While fitting a chevron (Main text Fig. 4b) can approximately find the resonance condition, calibrating the system to the degree required for fidelities above 99.9% requires more precise protocols. Using a four-wave mixing process to engineer the beamsplitter interaction also incurs drive-induced Zeeman shifts of the cavity frequencies that are on the same order as the beamsplitter rate, further complicating the calibration. If these shifts were identical for Alice and Bob, then the beamsplitting resonance condition would not change, but any relative difference between these shifts will manifest as a shift in the beamsplitting resonance condition. Most notably, this means that the resonance condition changes as a function of drive amplitude, complicating the process of calibrating a pulse with finite ramps.

Within the dual-rail qubit, Eq. 18 in the main text reduces to

$$\begin{aligned}\hat{\mathcal{H}}_{\mathrm{BS}}/\hbar &= ((\Delta_{ab} - \Delta_d) + \Delta_{\mathrm{Z},ab}\varepsilon(t))\frac{\hat{\sigma}_Z}{2} \\ &\quad + g_{\mathrm{BS}}\;\varepsilon(t)\;(e^{i\varphi_{\mathrm{BS}}}\hat{\sigma}_+ + e^{-i\varphi_{\mathrm{BS}}}\hat{\sigma}_-),\end{aligned}$$

where $\varepsilon(t) \in [0,1]$ is a normalized envelope function corresponding to the square of the pulse shape, as both pulses are ramped simultaneously.

In this Bloch sphere picture, the polar angle $\theta(t)$ of a state starting at the North Pole is given by

$$\theta(t) = 2\int_0^t g_{\mathrm{BS}}\;\varepsilon(t')dt'.$$

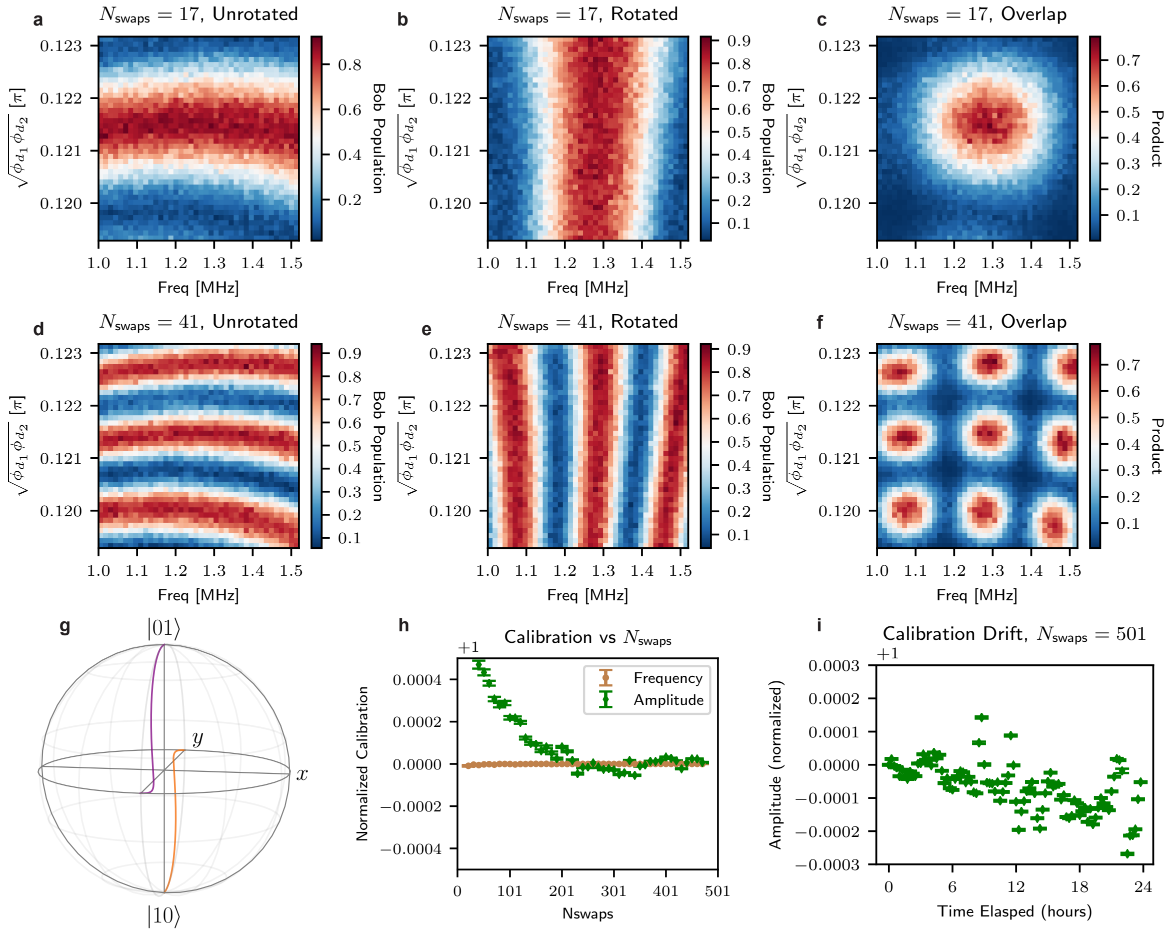

Fig. S6 Beamsplitter Pulse Calibration. **a**, Sweeping drive amplitude and detuning, we perform $2N_{\text{swaps}}$ consecutive beamsplitter pulses with identical phase, leading to bands that correspond to plausibly correct calibration values. **b**, By performing the same number of beamsplitter pulses as (a), but changing the phase of every two beamsplitters relative to the prior two, we can effectively engineer a sequence equivalent to $U_{\text{SWAP}}Z(\varphi)U_{\text{SWAP}}\ldots Z(\varphi)U_{\text{SWAP}}$. For a value of $\theta_R = 1.3$ radians (empirically found), we recover bands that now trace paths roughly perpendicular to those found from the (**a**) protocol. **c**, The correct calibration setting would pass both protocols, so we take the overlap, or product, of (**a**) and (**b**) to recover a region that the correct calibration is in. **d-f,** By increasing N_{swaps}, the bands get tighter individually, but also more densely spaced. This leads to multiple regions in the overlap plot (**f**), but only one region has the same center as regardless of N_{swaps}, in this case the center region. By iteratively narrowing the scope and increasing N_{swaps}, we can refine our calibration ever finer. **g,** Trajectories of states initialized on the poles of the dual-rail Bloch Sphere for the correct calibration. The pulse we calibrate for is detuned from the coherent evolution resonance condition, but the detuning combines with Zeeman shifts during the ramps (which appear as Z rotations on the Bloch sphere) to perfectly compensate and give the desired unitary. **h,** Calibration vs N_{swaps}. At each value of N_{swaps}, we fit the overlap region to a 2D Gaussian and extract center frequency (brown) and amplitude (green) as well as the fit error. By dividing these values by the final drive tone detuning ($\Delta_d = 237.26367$ MHz $\pm$ 50 Hz) and final amplitude ($\sqrt{\phi_{d_1}\phi_{d_2}} = 0.1214367 \pm 4 \times 10^{-7}$) respectively, we can plot the calibration normalized to the final parameter set as a function of N_{swaps} **i,** To track the significance of calibration drifts, we run the protocol for $N_{\text{swaps}} = 501$ every 15 minutes over a 24 hour period, finding no more than a 0.03% fluctuation over this timescale. The frequency calibration has no observable drifts.

The azimuthal phase drift during the operation is then given by

$$\delta\phi(t) = \int_0^t \sin(\theta(t'))\frac{((\Delta_{ab} - \Delta_d) + \Delta_{Z,ab}\,\varepsilon(t'))}{2}\,dt' \tag{24}$$

There exists a static detuning Δ_d such that, at a time τ_{BS} given by $\theta(\tau_{\text{BS}}) = \frac{\pi}{2}$ we can have complete cancellation of the azimuthal phase drift

$\delta\phi(\tau_{BS}) = 0$. Such a pulse takes the trajectory of a detuned process during the steady state before arriving back to the correct final position on the Bloch sphere during the ramp down (Fig. S6g).

To find the correct parameter set that achieves this, we can prepare a single photon in Bob, apply a candidate beamsplitter pulse a total of $4n + 2$ times, and measure $\text{P(Bob} = 0)$ to see whether the photon is successfully swapped out of Bob. Specifically, we fix the total pulse time and envelope shape while simultaneously sweeping tone 1 drive frequency (and thus Δ_d) and drive strength (and thus g_{BS} and $\Delta_{Z,ab}$) of the candidate pulse to scan the parameter space. If the candidate pulse does indeed implement a beamsplitter, then we will have implemented a total of $N_{swaps} = 2n + 1$ swap operations on the photon, leaving Alice with the photon and Bob in vacuum.

To gain some insight as to what we should expect when sweeping these parameters, we consider an adapted Rabi model with an amplitude-dependent detuning. Under this model, we find the probability $P_{B\to A}$ to oscillate as:

$$P_{B\to A} \approx \sin^2\left(2\sqrt{\Omega^2 + (\Delta_0 + \alpha\Omega)^2}t\right),$$

where Ω and Δ_0 are drive strength and detuning respectively, and α is some coefficient relating drive strength to frequency shift. For a fixed time T, we would expect contours of constant $P_{B\to A}$ to be of the form:

$$\Omega^2 + (\Delta_0 + \alpha\Omega)^2 = \left(\frac{(2n+1)\pi}{4T}\right)^2.$$

This equation, when varying Ω and Δ_0, results in ellipses with different bands corresponding to different values of n. As seen in Fig. S6a,d, a sweep of the parameter space does indeed recover these bands, with different bands corresponding to different odd integer fractions of the correct band. For example, a sequence that used (4(5)+2) pulses but only implemented 3 total swaps would produce a band at $\frac{3}{5}$ of the radius of the correct band.

To narrow down precisely where along these bands the correct detuning to properly null Eq. 24 is, we run a complementary pulse sequence. This complementary sequence consists of pairs of candidate beamsplitter pulses, with the phase φ_{BS} of each pair shifted by an angle θ_R relative to the previous pair. This relative angle between pulse pairs results in a rotation of the bands as shown in Fig. S6b,e, and a value of $\theta_R = 1.3$ radians makes the new bands approximately orthogonal to the unrotated version. The correct set of calibration parameters successfully swaps the photon under both of these pulse sequences, so by taking the overlap (product) of these 2D sweeps we can get patches of possible parameter sets as seen in Fig. S6c,f.

Since the density of the contour lines is set by the number of pulses in the sequence, increasing N_{swaps} not only makes the bands thinner individually, but also makes them appear closer together. Putting all of this together, we can start at a low value of N_{swaps} where the bands are spaced far apart and the overlap region is wide. Then, by refining the parameter range and increasing N_{swaps}, we can iteratively make the overlap region smaller and tighten the bounds on the correct amplitude and detuning. Since this scheme amounts to detecting the center of a circle in an image, it is amenable to basic image processing techniques, allowing an automation of the calibration procedure. We use this to calibrate the beamsplitter pulse up to ∼1000 operations, limited by FPGA wave memory, leading to a fractional calibration precision of less than 3×10^{-6} in Amplitude and 3×10^{-7} in frequency (Fig. S6h).

To monitor calibration drifts, the protocol was performed at $N_{swaps} = 501$ at 15 minute intervals for 24 hours (Fig. S6i). We observe no significant drift in frequency, while observing a drift of no more than 0.03% in amplitude. This amplitude drift, plausibly due to temperature fluctuations of the IQ-mixers and other control electronics, would only have a quadratic effect on the infidelity.

Supplementary Note 9 — Randomized Benchmarking protocols

Randomized benchmarking on a qubit encoded in a higher dimensional system presents unique challenges, particularly when leakage out of the qubit subspace is the dominant error [21]. However, the dual-rail qubit [22] is still amenable to RB-like techniques because this leakage to vacuum can predominantly be detected and selected out.

To carry out a randomized benchmarking protocol on the dual-rail qubit, we construct the gate

set G_{DR} with the following mapping:

$$\{X_{\pi/2}, Y_{\pi/2}, X_{-\pi/2}, Y_{-\pi/2}\} \rightarrow \{U_{\mathrm{BS}}(0), U_{\mathrm{BS}}(\pi/2), U_{\mathrm{BS}}(\pi), U_{\mathrm{BS}}(-\pi/2)\},$$

$$\{X_\pi, Y_\pi\} \rightarrow \{U^2_{\mathrm{BS}}(0), U^2_{\mathrm{BS}}(\pi/2)\}.$$

Here, all $U_{\mathrm{BS}}(\varphi)$ are generated by the same calibrated pulse (Methods 6.6), with varying drive phases (main text, Fig 3d). Only the relative difference between the pulse phases matter, which the FPGA-based control can guarantee with high precision, and we expect this phase's accuracy to be significantly higher than that of the amplitude and detuning of the pulse. We also purposefully exclude explicit Z gates from our gate set, as these gates could be achieved by a phase update on subsequent gates and are independent of the beamsplitter fidelity.

We generate sequences of up to 8100 such gates, by picking up to 900 random gates from G_{DR} in real time and repeating each gate 9 times consecutively. The impetus for this unique choice of concatenation is due to an FPGA memory limit of ~ 900 consecutive random operations. By re-defining one 'operation' to be $4n + 1$ concatenations of the same gate (utilizing the fact that $g^{4n+1} = g, \forall g \in G_{\mathrm{DR}}, \forall n \in \mathbb{N}$) we can go beyond this limitation to capture longer timescales. At each sequence length, we generate 10^5 such semi-random sequences for adequate precision even after post-selection. These gates form an over-complete set of generators for the Clifford group, which can be seen by constructing the dual-rail Hadamard and S gates:

$$H_{\mathrm{DR}} = X_\pi Y_{\pi/2} \tag{25}$$

$$S_{\mathrm{DR}} = Y_{-\pi/2} X_{\pi/2} Y_{\pi/2}. \tag{26}$$

Each sequence maps the initial state $|0_a 1_b\rangle$ to a known cardinal point on the Bloch sphere, after which we apply a single additional gate $U^{-1}(\varphi_n)$ that brings the state back to $|0_a 1_b\rangle$. This state-mapping inverse differs from a full unitary inverse by up to a Z rotation, but since the cavity measurements correspond to a logical Z measurement of the dual-rail qubit, the outcomes are unaffected by this difference.

This protocol is similar to 'Direct RB' [23], with three notable exceptions - the leakage out of the subspace due to single-photon loss errors, the repeated gates, and the difference in the choice of the final Unitary. Since the leakage is a detectable error, it can be separately selected out and quantified, leaving sequences that are fully randomized to a depolarization channel under a sufficiently large number of averages and sufficiently deep circuit ($\sim 10^4$ averages for the first 2250 gates), assuming effects due to a difference in the cavity decay rates is negligible (see Table S1 for coherences). We expect the repeated gates to slightly enhance the effect of systematic errors, like those in our calibration, but still result in depolarization over sufficiently long timescales. We explicitly check this by comparing a more conventional non-repeated gate RB to the decay given by a repeated protocol of sets of 5 gates (Fig. S7a). Finally, our dispersive measurement scheme's errors should only depend on the expectation value of Z, and thus the extra Z rotation in our inverse should not affect to the protocol. We thus expect the arguments in [23] to hold for our post-selected protocol.

We first execute this protocol with no selection (other than on the final state of Bob's ancilla) and measure the success probability $P_{0_a 1_b}$ of returning the system to its initial state. In general, this curve should have two characteristic timescales corresponding to decay out of and dephasing within the dual-rail subspace. However, as a sign of our strong noise-bias towards photon-loss errors, we find that the probability decays in a dominantly single-exponential manner (Fig. S7), with a decay constant of $\tau_{\mathrm{RB}} = 1271 \pm 4$ gates. We use this exponential behaviour to estimate an effective average unselected gate infidelity of $0.078 \pm 0.001\%$.

To accurately quantify the rate of decay, we use our measurements of Alice to find the combined probability of staying in the dual-rail subspace after each sequence, $P_{1_a 0_b \,\cup\, 0_a 1_b}$. This probability is unaffected by dephasing within the subspace, allowing us to extract a pure leakage timescale of $\tau = 1388 \pm 4$ gates, corresponding to a $0.072 \pm 0.001\%$ probability of decay per gate. It is clear that our 'raw' RB is strongly limited by this timescale, given the overlap of the two curves in Fig. S7.

Finally, we focus on the error-detected dataset, containing sequences with no leakage events (we ignore the minute probability of both a cavity decay and heating event occurring within these

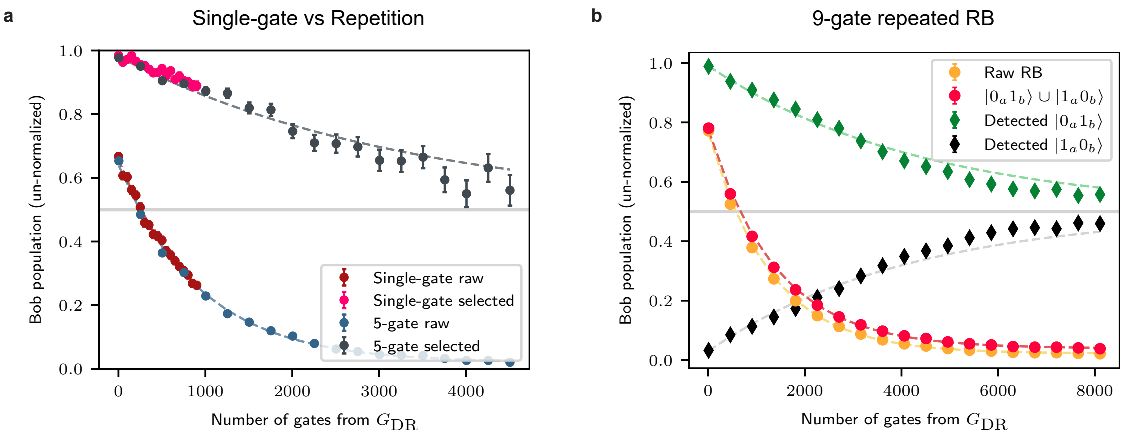

Fig. S7 **Randomized Benchmarking protocols. a,** Comparison of a standard RB protocol (each gate random) to a protocol with random gates repeated in sets of five. The un-selected (raw) data agree within error-bars. On post-selecting out single-photon loss events, the five-gate sequence provides a lower bound for the fidelity of the single-gate sequence, since repeating sets of gates amplifies the effects of coherent errors, like those due to miscalibration. **b,** We analyze in detail a single RB experiment with random sets of 9 repeated gates, under various selection protocols, with no normalization. Dashed lines for each curve represent exponential fits. The raw RB (yellow) shows a decay constant of 1271 ± 4 gates, with its reduced amplitude corresponding to a SPAM error of $\sim 21\%$. The leakage-limited curve (red) represents the total rate of photon loss out of the dual-rail subspace, and does not distinguish between states on the dual-rail Bloch sphere. This curve has large overlap with the raw RB, implying that we are limited by photon loss errors, and shows a decay constant of 1388 ± 4 gates. On detecting and selecting out the leakage events, we obtain error-detected curves where the system ended in either $|0_a1_b\rangle$ (green) or $|1_a0_b\rangle$ (black). These sequences on average represent a depolarization channel, and seem to decay towards a perfectly mixed state (0.5, grey horizontal line) with improved decay times of 4477 ± 30 and 4194 ± 32 gates respectively. All exponential fits take standard errors (SE) of each dataset into account, with data-points with fewer averages contributing less to the fit, which is particularly visible in the error-detected datasets.

timescales). These sequences contain trajectories which remain in the dual-rail subspace, with errors that arise from driven dephasing, 'no-jump' evolution towards the higher coherence cavity [22], and imperfect control. As argued above, we expect these errors to be converted to a depolarization channel under the RB protocol. Fitting these curves with an exponential decay to an offset of 0.5, we extract time constants of $\tau = 4477 \pm 30$ gates and $\tau = 4194 \pm 32$ gates for the error-detected $P_{0_a1_b}$ and $P_{1_a0_b}$ data, respectively.

Notably, the error-detected curves deviate from an exponential fit at long timescales. This could be due to a resurgence in ancilla-induced dephasing at timescales where the ancilla can both heat and decay, or significant probability that sequences containing photons at long timescales arise from cavity heating instead of the initial state preparation, or minor imperfections in state readout that are only visible when Alice and Bob populations are comparable. For these reasons, we restrict our fits in the main text to the first 2250 gates, where the decay is well-described by an exponential decay, and obtain an error-detected infidelity of $0.02 \pm 0.001\%$. Finally, since each gate in G_{DR} is constructed from one or more near-identical beamsplitter pulses, we use the fact that the average gate has 4/3 beamsplitters to convert this gate infidelity into an error-detected beamsplitter infidelity of $0.015 \pm 0.001\%$. This factor of 4/3 comes from the fact that 4/6 gates in G_{DR} are beamsplitter pulses and 2/6 gates are swap operations implemented with two consecutive beamsplitters. This leads to an average of $1 \times 4/6 + 2 \times 2/6 = 4/3$ beamsplitters per gate on average in G_{DR}.

Supplementary Note 10 — Data normalization

We detail in this section any schemes used to normalize data in the main text, which were used to put focus on the relative decay of fidelity as a function of time or number of gates, instead of SPAM errors. The coherent evolution data shown in Fig. 2b of the main text was normalized with respect to a separately measured readout infidelity. This measurement involved executing the

standard protocol for preparing a single photon in Bob (described in the main text) and probing its state through selective spectroscopy of Bob's ancilla. The resulting number-split peaks showed population at $|0_b\rangle$ and $|1_b\rangle$ respectively, with no measureable population at Fock states $|2_b\rangle$ and higher. A simple linear transformation was then applied to the spectroscopy data such that the offset was set to zero, and the total population in $|0_b\rangle$ and $|1_b\rangle$ exactly summed to one. The renormalized population in $|1_b\rangle$ was inferred to be the state preparation fidelity ($\sim$ 94%). An identical linear transform was applied to the coherent evolution data.

The RB datasets (shown in Fig. 3 of the main text) were normalized in different ways. The raw and the coupler-selected datasets were both exactly normalized to span between 1.0 (at zero gates) and 0.0 (at 8100 gates), to ignore SPAM errors. The error-detected dataset was normalized such that it had a value of 1.0 at zero gates, and a steady-state value of 0.5 would map to a normalized value of 0.0. This steady-state estimate of 0.5 was chosen due to a lack of high-confidence data points at very long times, to allow a visual comparison of the error-detected dataset to the raw dataset. All unnormalized RB curves are shown in Fig. S7.

Supplementary Note 11 — Device Fabrication

We detail here the fabrication of the superconducting circuits used in our experiment. The coupler and the ancilla transmon chip devices are fabricated with e-beam lithography on a 2-inch, 430 µm thick C-plane EFG sapphire substrate. E-beam writing of the Josephson junctions (designed in the 'Manhattan' style with 90° cross-over), as well as the coarser patterns, is performed in Raith EBPG 5000+. Two aluminum layers are deposited via dual-angle electron-beam evaporation in a Plassys UMS 300, and are gapped by an Al_xO_y insulator layer grown in an oxidation process under 20 mbar of 85% Ar and 15% O_2 mixture for 12 minutes. For both chip devices, two adjacent stripline resonators with meandering-line geometry are used as the on-chip readout mode and Purcell filter mode (Fig. S8), with the readout mode dispersively coupled to the coupler/ancilla mode, and the Purcell filter mode strongly coupled to the readout port. The superconducting package hosting the chips is made of a single piece of high-purity 99.999% ('5N') Aluminum and is chemically etched to improve surface quality.

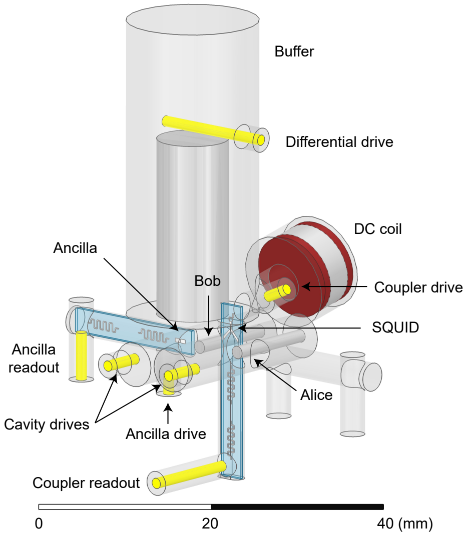

Fig. S8 Illustration of the full package. An x-ray view showing the relative orientations of the buffer cavity, coupler, Alice, Bob, the ancilla and the electromagnetic coil used for DC flux delivery. The non-reflective grey space is vacuum, and the grey co-axial posts in the cavities are high-purity Aluminum. Alice, Bob and the buffer mode are the three coaxial stub cavities with designed stub lengths of ≈11.2mm, 10.7mm and 21.4mm, respectively. Both the coupler and the ancilla have on-chip stripline resonators that act as Purcell filters and readouts respectively. Yellow rods denote capacitively coupled transmission line 'pins' that are used to address each mode.

Supplementary Note 12 — Device Parameters

We display in Tables S1 and S2 the measured device parameters of the SQUID coupler, Alice, Bob, and Bob's transmon ancilla. Here, T_2^* and T_2^E are extracted from Ramsey and T_2 Echo experiments, respectively. The Coupler T_1 has been observed to drift between 40 and 80μs within and between cooldowns. Bob's diminished coherence times as compared to Alice are likely to do the smaller detuning between Bob and the Coupler, leading to increased participation in the bare coupler mode, as well as deleterious effects of the Transmon ancilla. In particular, Bob's T_2 likely suffers due to the relatively high thermal population (and thus heating rate) of the ancilla.

Cross-Kerrs between the ancilla and the distant Alice and coupler modes were not directly measured, but are assumed to be negligible. From the cross-Kerrs measured between the coupler and the storage cavities, we infer their direct linear coupling strengths to be $g_{ac}/2\pi \approx 82$ MHz, and $g_{bc}/2\pi \approx 78$ MHz.

	Coupler	Alice	Bob	Ancilla
Frequency (GHz)	7.245	6.225	6.46	5.663
T_1 (μs)	~60	375	300	120
T_2^* (μs)	25-35	450	250	5.5
T_2^E (μs)	40	N/A	N/A	25
n_{th}	0.02	<0.03	<0.03	0.06

Table S1 Measured frequencies, coherence times, and thermal populations for the SQUID's coupler mode, the storage modes Alice and Bob, and Bob's coupled ancilla transmon.

Kerrs	Coupler	Alice	Bob	Ancilla
Coupler	-125 MHz	-1.7 MHz	-2.6 MHz	N/A
Alice		-4.9 KHz	-11 KHz	N/A
Bob			-14.6 KHz	-1.2 MHz
Ancilla				-180 MHz

Table S2 Measured self-Kerrs and cross-Kerrs for the four modes. The cross-Kerr between the ancilla and the coupler or Alice modes have not been measured, but are assumed to be negligible.

Supplementary Note 13 — Wiring Diagram

The schematic of the instrumentation and cryogenic setup can be seen in Fig. S9. The coupler and the ancilla transmon chip devices are held by chip clamps that are bolted to the superconducting Aluminum package. The package is then heat sunk to the base stage (at ~20 mK) of an Oxford Instruments dilution refrigerator via an OFHC copper post. A Cryoperm can surrounds the package and provides magnetic shielding, with an inner layer of Berkeley-black-coated copper shim (not shown) acting as an IR photon absorber. seven microwave lines with different filtering schemes connect the package to the room-temperature (RT) setup: the drive lines and the readout lines for preparing and reading out the states of the coupler (common mode) and the ancilla, the drive lines for displacing Alice and Bob, and a buffer-mode drive line for applying the two RF flux drives.

To maintain the phase stability of the beam-splitter pulse relative to the cavity drives, three local oscillators (LOs, Agilent N5183A RF signal generators) are shared between Alice, Bob and the buffer mode, as indicated in Fig. S9. The down-conversion LO ($\omega_\Delta = 9.2$ GHz) mixes with the Alice LO ($\omega_a = 6.29$ GHz) and the Bob LO ($\omega_b = 6.525$ GHz), creating two drive tones at $\omega_{d_2} = 2.91$GHz and $\omega_{d_1} = 2.675$GHz that serve as effective LOs for the two flux drives. The Coupler, ancilla and readout drives are created by independent LO sources. The LO tones are then mixed with waveform pulses generated by four FPGA-based quantum controllers (Innovative Integration X6-1000M). The custom-built DC flux line carries the dc current generated by a YOKOGAWA GS200 low-noise voltage source that we use to bias the SQUID to true zero flux quantum. The DC flux line is made of copper from RT to 4K, soldered to NbTi superconducting wire from 4K to below, with the solder joint thermalized to the 4K plate to ensure that the thermal dissipation does not exceed the cooling power of the dilution refrigerator. Measurement of the coupler and the ancilla is done with dispersive readout in reflection. The readout signals are amplified by two SNAIL parametric amplifiers (SPA) at the base stage, and are further amplified by HEMT amplifiers at 4 K and MITEQ amplifiers at room temperature, then down-converted to an intermediate frequency (50 MHz) by the same readout LO, and digitized by a pair of ADCs integrated into the FPGA cards.

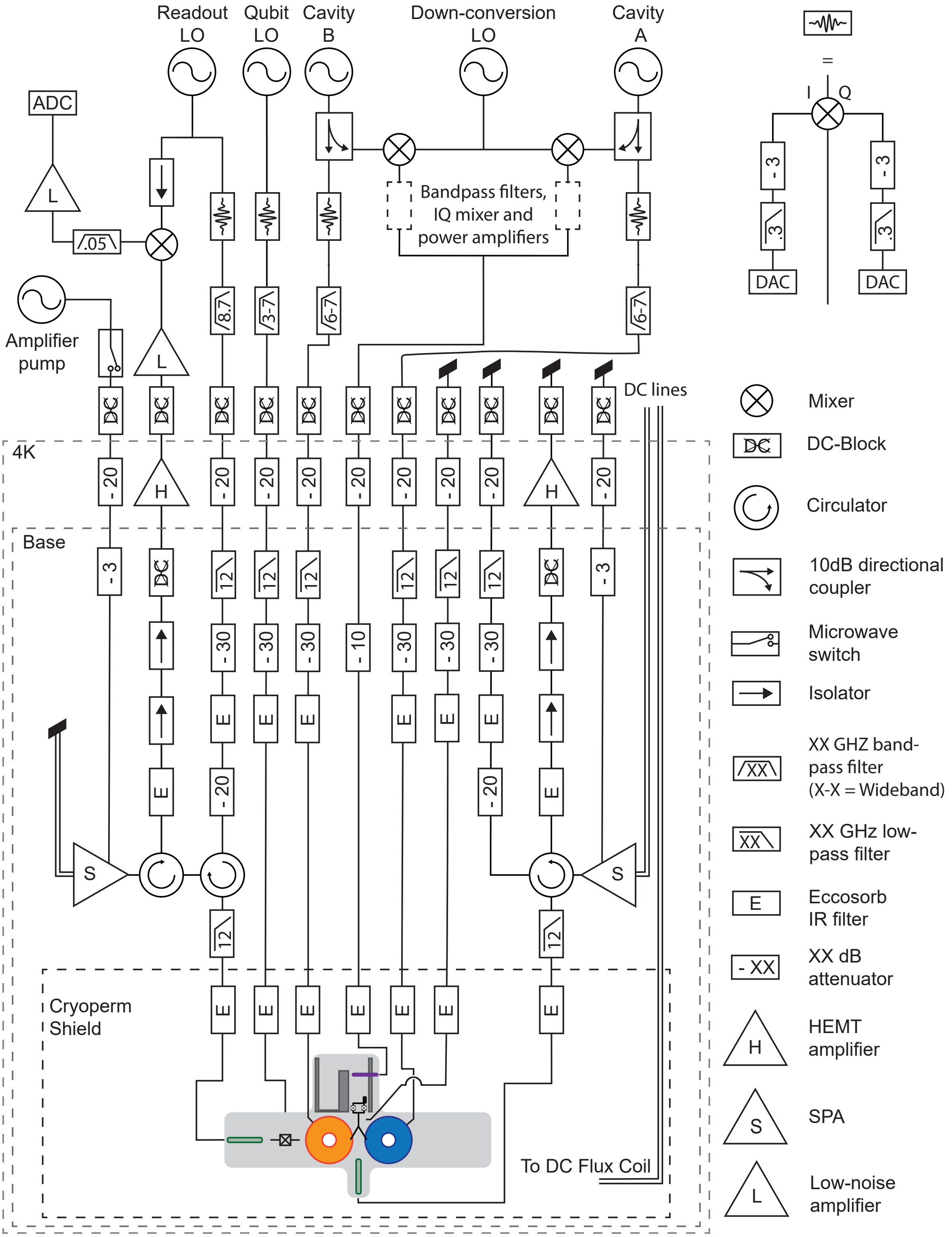

Fig. S9 **Wiring Diagram**

References

[1] Lecocq, F., Claudon, J., Buisson, O., Milman, P.: Nonlinear coupling between the two oscillation modes of a dc squid. Phys. Rev. Lett. **107**, 197002 (2011). https://doi.org/10.1103/PhysRevLett.107.197002

[2] Kamal, A., Clarke, J., Devoret, M.H.: Gain, directionality, and noise in microwave squid amplifiers: Input-output approach. Phys. Rev. B **86**, 144510 (2012). https://doi.org/10.1103/PhysRevB.86.144510

[3] Gao, Y.Y., Lester, B.J., Zhang, Y., Wang, C., Rosenblum, S., Frunzio, L., Jiang, L., Girvin, S.M., Schoelkopf, R.J.: Programmable interference between two microwave quantum memories. Phys. Rev. X **8**, 021073 (2018). https://doi.org/10.1103/PhysRevX.8.021073

[4] Kohler, S., Dittrich, T., Hänggi, P.: Floquet-markovian description of the parametrically driven, dissipative harmonic quantum oscillator. Phys. Rev. E **55**, 300–313 (1997). https://doi.org/10.1103/PhysRevE.55.300

[5] Verney, L., Lescanne, R., Devoret, M.H., Leghtas, Z., Mirrahimi, M.: Structural instability of driven josephson circuits prevented by an inductive shunt. Phys. Rev. Appl. **11**, 024003 (2019). https://doi.org/10.1103/PhysRevApplied.11.024003

[6] Zhang, Y., Lester, B.J., Gao, Y.Y., Jiang, L., Schoelkopf, R.J., Girvin, S.M.: Engineering bilinear mode coupling in circuit qed: Theory and experiment. Phys. Rev. A **99**, 012314 (2019). https://doi.org/10.1103/PhysRevA.99.012314

[7] Shillito, R., Petrescu, A., Cohen, J., Beall, J., Hauru, M., Ganahl, M., Lewis, A.G.M., Vidal, G., Blais, A.: Dynamics of transmon ionization. Phys. Rev. Appl. **18**, 034031 (2022). https://doi.org/10.1103/PhysRevApplied.18.034031

[8] Yan, F., Campbell, D., Krantz, P., Kjaergaard, M., Kim, D., Yoder, J.L., Hover, D., Sears, A., Kerman, A.J., Orlando, T.P., Gustavsson, S., Oliver, W.D.: Distinguishing coherent and thermal photon noise in a circuit quantum electrodynamical system. Phys. Rev. Lett. **120**, 260504 (2018). https://doi.org/10.1103/PhysRevLett.120.260504

[9] Minev, Z.K., Leghtas, Z., Mundhada, S.O., Christakis, L., Pop, I.M., Devoret, M.H.: Energy-participation quantization of josephson circuits. npj Quantum Information **7**(1) (2021). https://doi.org/10.1038/s41534-021-00461-8

[10] Clerk, A.A., Devoret, M.H., Girvin, S.M., Marquardt, F., Schoelkopf, R.J.: Introduction to quantum noise, measurement, and amplification. Rev. Mod. Phys. **82**, 1155–1208 (2010). https://doi.org/10.1103/RevModPhys.82.1155

[11] Strand, J.D., Ware, M., Beaudoin, F., Ohki, T.A., Johnson, B.R., Blais, A., Plourde, B.L.T.: First-order sideband transitions with flux-driven asymmetric transmon qubits. Phys. Rev. B **87**, 220505 (2013). https://doi.org/10.1103/PhysRevB.87.220505

[12] Heeres, R.W., Vlastakis, B., Holland, E., Krastanov, S., Albert, V.V., Frunzio, L., Jiang, L., Schoelkopf, R.J.: Cavity state manipulation using photon-number selective phase gates. Phys. Rev. Lett. **115**, 137002 (2015). https://doi.org/10.1103/PhysRevLett.115.137002

[13] Zhang, Y., Curtis, J.C., Wang, C.S., Schoelkopf, R.J., Girvin, S.M.: Drive-induced nonlinearities of cavity modes coupled to a transmon ancilla. Phys. Rev. A **105**, 022423 (2022). https://doi.org/10.1103/PhysRevA.105.022423

[14] Johansson, J.R., Nation, P.D., Nori, F.: QuTiP: An open-source python framework for the dynamics of open quantum systems. Computer Physics Communications **183**(8), 1760–1772 (2012). https://doi.org/10.1016/j.cpc.2012.02.021

[15] Campagne-Ibarcq, P., Eickbusch, A., Touzard, S., Zalys-Geller, E., Frattini, N.E., Sivak, V.V., Reinhold, P., Puri, S., Shankar,

S., Schoelkopf, R.J., Frunzio, L., Mirrahimi, M., Devoret, M.H.: Quantum error correction of a qubit encoded in grid states of an oscillator. Nature **584**(7821), 368–372 (2020). https://doi.org/10.1038/s41586-020-2603-3

[16] Sivak, V.V., Eickbusch, A., Royer, B., Singh, S., Tsioutsios, I., Ganjam, S., Miano, A., Brock, B.L., Ding, A.Z., Frunzio, L., Girvin, S.M., Schoelkopf, R.J., Devoret, M.H.: Real-time quantum error correction beyond break-even. Nature **616**(7955), 50–55 (2023). https://doi.org/10.1038/s41586-023-05782-6

[17] Ofek, N., Petrenko, A., Heeres, R., Reinhold, P., Leghtas, Z., Vlastakis, B., Liu, Y., Frunzio, L., Girvin, S.M., Jiang, L., Mirrahimi, M., Devoret, M.H., Schoelkopf, R.J.: Extending the lifetime of a quantum bit with error correction in superconducting circuits. Nature **536**(7617), 441–445 (2016). https://doi.org/10.1038/nature18949

[18] Gertler, J.M., Baker, B., Li, J., Shirol, S., Koch, J., Wang, C.: Protecting a bosonic qubit with autonomous quantum error correction. Nature **590**(7845), 243–248 (2021). https://doi.org/10.1038/s41586-021-03257-0

[19] Ni, Z., Li, S., Deng, X., Cai, Y., Zhang, L., Wang, W., Yang, Z.-B., Yu, H., Yan, F., Liu, S., Zou, C.-L., Sun, L., Zheng, S.-B., Xu, Y., Yu, D.: Beating the break-even point with a discrete-variable-encoded logical qubit. Nature **616**(7955), 56–60 (2023). https://doi.org/10.1038/s41586-023-05784-4

[20] Ambegaokar, V., Baratoff, A.: Tunneling between superconductors. Phys. Rev. Lett. **10**, 486–489 (1963). https://doi.org/10.1103/PhysRevLett.10.486

[21] Wood, C.J., Gambetta, J.M.: Quantification and characterization of leakage errors. Phys. Rev. A **97**, 032306 (2018). https://doi.org/10.1103/PhysRevA.97.032306

[22] Teoh, J.D., Winkel, P., Babla, H.K., Chapman, B.J., Claes, J., de Graaf, S.J., Garmon, J.W.O., Kalfus, W.D., Lu, Y., Maiti, A., Sahay, K., Thakur, N., Tsunoda, T., Xue, S.H., Frunzio, L., Girvin, S.M., Puri, S., Schoelkopf, R.J.: Dual-rail Encoding with Superconducting Cavities. https://arxiv.org/abs/2212.12077

[23] Proctor, T.J., Carignan-Dugas, A., Rudinger, K., Nielsen, E., Blume-Kohout, R., Young, K.: Direct randomized benchmarking for multiqubit devices. Phys. Rev. Lett. **123**, 030503 (2019). https://doi.org/10.1103/PhysRevLett.123.030503
